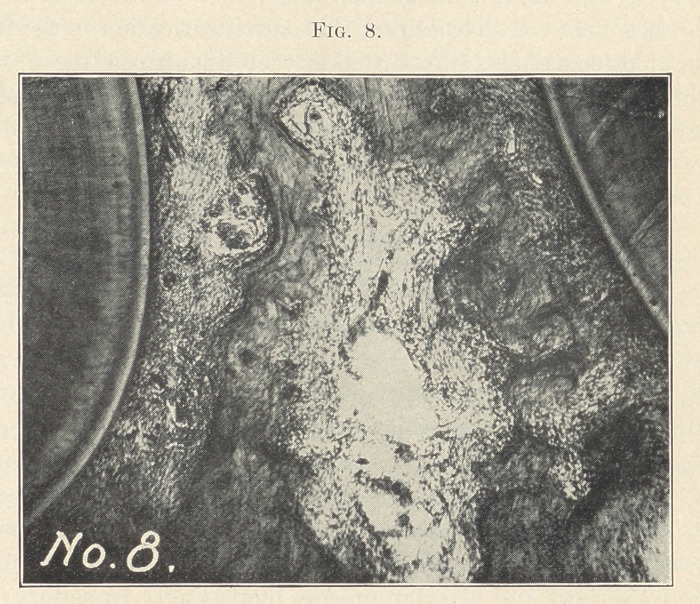# Reviews of Dental Literature

**Published:** 1905-01

**Authors:** 


### Reviews of Dental Literature.

---

The Choice and Use of Medical Literature.[1] By Hugh T. Patrick, M.D., Chicago.

The choice and the use of medical literature depend first on what the physician tries to be; not on what he would like to be, not on what his ideal may be, but on what he thoughtfully, consistently strives to be.

KINDS OF PHYSICIANS.

Our day-dreams of attainment are much the same, but the daily walk of physicians varies greatly. Most of us have met a physician who may be called the family factotum. He hobnobs with fussy mothers and puttering fathers. He is greatly interested in grandma's cough, knows just how to wash the baby, has his special poultice, and can take off warts. Dropping in to ask about Aunt Em's backache, he stays an hour visiting with the folks. He is a gentle and kindly soul, but his mind is occupied with the trivialities of medicine and domestic chit-chat.

Then, there is our old friend who in winter airs his surgical deeds and medical acumen about the drug-store stove and in summer holds a like symposium on the shady side of the street. He knows a good cigar, is a pleasant gentleman, and harmless in all things save only the practice of his profession.

We have heard of the affable society physician who dresses well, talks well, knows the best families, attends receptions, and makes social calls. We know and respect the God-fearing church physician who teaches the Bible-class, sings in the choir, gets together the pastor's salary, and finances the church debt. The lodge physician, the sporting physician, and the physician deep in politics are familiar figures. We know them all. They are of us, members of our honorable guild. With loyalty and affection we take

---

[1] President's address, delivered before the thirtieth annual meeting of the Mississippi Valley Medical Association, at Cincinnati, October 11, 1904. [This address is equally applicable to dentists, and its forcible statements should be read and appreciated.—Ed.]

them by the hand. They are good friends and good citizens; but really fine physicians—not one of them. And not one of them has any real use for good medical literature.

But there is another man—the man we would like to be; the capable man who knows his work and does it; the man up with the van, who can talk face to face with the great in our noble profession. He has enthusiastically striven or doggedly persisted until he is the wise, skilful physician who practises with no uncertain hand, but knows when he is right and knows where he is ignorant. To be what he is and do what he does means familiarity with what others have done and are doing. To this there is no exception. He reads. He selects his literature well and uses it wisely.

### WISE SELECTION OF BOOKS.

The selection of our books is a task as delicate, a duty as significant as is the choice of remedies for our patients. Every book is a prescription for our mental self. And yet, who can deny that the clever book-agent puts thousands of volumes on our shelves? In this day and generation it is the physician's obligation not only to be able to recognize the good in medical literature, but to know the great and the safe among medical writers. A conscientious physician calls in consultation no unknown man, but, strange to relate, he will blindly follow an author of whom he knows nothing except that he has produced a book.

### THE ROLE OF THE TEXT-BOOK.

Another somewhat frequent professional indiscretion, to call it by no harsher name, is the good old student plan of sticking to a text-book. Progress means expansion. The more a man progresses the less prominent is the rôle of the text-book. It is necessary for babes, but not meat for strong men. No text-book physician is an A No. 1 man. The latter needs the treatise, the "system," the cyclopædia, and particularly the monograph.

### THE MONOGRAPH.

Of all medical books, the monograph is the best—and the worst. As a rule, it is the product of special interest and the wise employment of opportunity. In it we often find the rich harvest of many years of loving labor by a great mind. In all probability, the author has not only consulted the writings of others, but has acquired a sure judgment in weighing their merits. Unfortunately, sometimes he is an enthusiast without balance, a faddist, a man with a theory, a prejudiced observer, a dealer in sophistry. Then, if in addition he be a positive and clever writer he may do untold harm. I have now in mind an interesting, attractive book which in the last twelve years has led astray thousands of physicians, and I am sure that the next quarter of a century will not see rooted out the fallacies planted by its brilliant author. Why is this? Because thousands of well-meaning physicians bought the book without knowledge, read it without care, absorbed its tenets without proving, and promulgated its dicta without prudence.

DISCRIMINATION IN READING.

If he is to really profit by the precious time spent in reading, the physician must be able to read with discrimination. In medicine there is no such thing as an authority. The critical sense must be keen and ever alert. The reader must learn to be a judge, for the plea of every paragraph is to be adjudicated. And if he feels himself at fault, assistance is on every side. I think there is no case in which those who know are more happy to lend a hand than in the selection and interpretation of medical writings.

MEDICAL JOURNALS.

And next, what of the journals? This is a big question and a hard one; one I approach with considerable feeling, but with no confidence. Nevertheless, I shall not attempt to dodge it; and since to advise is more human than to confess, to find fault more spontaneous than to praise, to say "don't" easier than to say "do," I venture first to advance a few of the "don'ts" in my mind. Be it understood, however, that these are suggestions purely tentative, as the expression of only one of a vast company, and that no remark is meant as criticism of editor, censure of publisher, or condemnation of publication *per se*. All is addressed to the reader. I am not talking of foods, but of diet.

Don't admit to your presence a journal that is not perfectly straight and clean. As the first requisites of the good physician are conscientiousness and strictest integrity, so no journal is safe if it be not honest on every page. Humiliating as is the admission, we must confess that there are mercenary medical men writing subsidized articles for the benefit of tradesmen. We may not be able to stop the practice, but for the love of decency, let us drop the articles into the waste basket as soon as received and bar the journals from our table.

Just to illustrate how vigilant we must be, I may state that one medical screed has appeared in different journals as a straight advertisement, an editorial, an "original," a therapeutic hint, a news item, and a clinical note. Again, of seven so-called original papers in one journal, four were obviously for ulterior purposes.

A thinly veiled deception is that of putting amidst scientific matter blatant advertising quoted from *Der Deutsche Medizinische Schurkenstreich* or *La Nation Médicale Trompeuse.* It is simply knavery with German or French sugar coating.

Another sort of debasing journal is the one that pretends, perhaps honestly, to help the physician to success by means other than pure professional excellence. A type of this kind of thing I recently found in an address to the graduating class of a medical school. I have no doubt the orator thought he was advising tact and inculcating practical methods for the management of patrons, the confusion of competitors, and the increase of income. As a matter of fact, he was teaching those young men deception, subterfuge, and meretricious connivance, to take the place of scientific knowledge and manly worth. Concerning the editor who spreads such pestilence, I have nothing to say. The physician who admits it to his library and his mind not only injures himself, but is recreant to his trust.

Don't indulge in yellow journals—for such there are—of deeper or fainter dye. The more harmless kinds merely present a sort of pseudoscientific vaudeville of striking oddities, rare curiosities, marvellous happenings, and other side-show monstrosities of the medical world. All of this serves only to excite a passing interest, and makes no reader one whit better as scholar or practitioner. The worse kinds, under the cloak of medicine, pander to our appetite for the startling, the scandalous, even the salacious, and appeal to passion and prejudice. They lean strongly to sexual perversion and suggestive gossip. Behind the mask of independent thought they delight in strictures on those high in the profession and even indulge in dirty innuendo. They start hot discussions on ethics and foment controversies over personal rights, privileges, and immunities. And this, God save the mark, is circulated as medical literature, because it is read by medical men.

Don't take a journal which is run as an advertising medium, and do not look at it if it is sent to you. Such publications are of two sorts. One kind is issued by some commercial gentleman to assist in selling his wares. In the guise of a scientific periodical, it is to all intents and purposes a sort of medical almanac, about as wholesome and edifying as the pamphlet for Bonesetter's bitters or the bulletin of Mother Udder's uterine uplifter. The other kind is conducted by a medical man with the dollar mark stamped on his aspirations. The good of the reader is no concern of his. He is after the money of advertisers, all of them. Original communications, editorials, excerpts, correspondence, everything, is arranged purely as a bait to induce the gullible to swallow the ads.

Don't read a journal that accepts abortive papers by undertone doctors. There are journals which systematically encourage that sort of thing. Unquestionably, to report cases and write down his opinions is good for any physician. But how about the reader? When the cases are incompletely studied, the writer ignorant or narrow, his conclusions lacking foundation, and his judgment immature, the contribution is not only valueless—it is injurious. Smooth is the descent that leads to Avernus, and easy the downward road of this damning third-rate literature. It is light and easy reading, but begets self-satisfaction, blunts the critical sense, lulls ambition, dulls observation, stunts mental growth, and before he knows it the reader is found on a low plane of thought and practice—no higher than the twaddle he reads.

Don't waste time on journals abounding in short cuts. They are an abomination unto the mind, a snare for the unwary, and their name is legion. A hint or two will indicate the sort I mean.

Purporting to be practical and immediately helpful, some journals make a specialty of what may be called recipes for disease. And they are very alluring. Instead of learning all about pneumonia, its nature, course, variations, and complications, what methods of treatment have been tried and abandoned and what has been the experience of those seeing hundreds of cases, it is so much easier to take some fellow's or some journal's statement that a peculiar poultice or ambitious alkaloid cures the disease. Stamped deep on my feelings is a paper on dyspepsia in a journal of great vogue. With no statement as to what dyspepsia may be, with no word as to diagnosis, with no allusion to pathology, no mention of gastroptosis, dilatation, hyperacidity, or motor power, and no hint of test meal or examination of stomach contents, the author proceeded to advise the administration of seven different drugs. In spite of the multifarious remedies, such advice simplifies practice to a degree. It is no task simply to remember to give this tonic for appetite, that capsule for digestion, this granule for pain, those drops for nausea, one pill for constipation, the other for diarrhœa, and the powder for flatulence. It is a short cut, but it leads to disaster.

In this same category belong the medical magazines that make a leader of questions and answers; a department modelled on the *Ladies' Fireside Guide,* where anxious inquiries go off at half-cock and the answers pop back as prompt and empty as echoes. A very little reflection will show not only the utter futility of this kind of reading, but how it prevents development by curtailing wholesome mental effort.

Then there is the petty journal corresponding to the family factotum above mentioned. Its short cut is simply the avoidance of the great and profound in medicine. Ignoring such fundamental things as anatomy, physiology, and pathology, oblivious alike to basic principles and the best of accumulated experience, it propagates a sort of family confab on the various superficialities of practice. How to bring out the eruption of measles; what is good for hiccough; the best liniment for sprains; these are cheerfully aired in numerous columns. What do you say to the parturient woman when she grows impatient? What is your favorite catarrh snuff? In the treatment of "threatened" appendicitis should aconite be given the first day and veratrum the second, or *vice versa?* On questions such as these, the editor and his writing readers expend great energy, priceless time, and endless ink. With great zest the contributors, as Charles Lamb says, encourage each other in mediocrity. I carefully went through one hundred and fifteen pages of such a penny-wise, pound-foolish publication, and found just six pages of good stuff. And yet that journal has an enormous circulation—to the great renown of the publishers and the great discredit of the medical profession.

Don't pay much attention to columns of formulæ, notes on treatment, therapeutic hints, brief paragraphs on recent discoveries, and items on new drugs. Pass over abstracts in which the process of condensation has squeezed the life out of the matter, and skip society reports so meagre as to amount to mere personal mention.

Most of such matter is garbled at the best, has no educational value, and, even when a bit of it has virtue, is pretty sure to just slip through the otic tunnel—in one ear and out the other.

From the foregoing negatives a few positives may readily be inferred. As we are to buy only the best books, so let us take only high-class medical periodicals. And then let us read them well. If there are many poor journals, there is much poor reading done; reading that is casual, unsystematic, careless, superficial, cursory, profitless. We have three sources of information and inspiration, —personal experience, personal contact with colleagues, and reading. The first may be limited, the second unsatisfactory, but the last offers to all the highest inspiration, and knowledge without end. Then let us read up and not down.

There is a certain comfortable ease in reading what we already understand. We may gratify our natural craving for approval by revelling in nice little papers which repeat what we have been saying for years. To tickle our vanity by reading papers so poor that they show the author to be more ignorant than ourselves is a pleasing process. Sometimes we feel luxuriously virtuous when reading a medical journal purely for entertainment and mental relaxation. None of these things should be. One and all they create a slovenly habit of mind. They are destructive of good method, and in the end incapacitate us for good work.

Here I must notice an objection or complaint that we have all heard and most of us have made: "I have no time to read." It is not true. The apportionment of time is not a matter of necessity, but of choice. What do I consider of the greater importance? What do I like and dislike? What do I choose to do? These are the determining questions. And in the modern physician's life there is precious little paramount to study. It pays to read. Besides the pleasure of knowledge and the power that knowledge gives, besides the gratifying sense of achievement and the satisfaction of progress toward a goal, it pays in dollars. In conversation with the busiest, the greatest, and the most successful of our colleagues, I have often been astonished at the amount of reading they do and how they rely on it. Very, very often good reading makes the difference between five dollars and twenty-five dollars for an examination, between fifty dollars and five hundred dollars for an operation.

And now, if I may be allowed four little hints as to the manner of reading, I shall have finished.

One excellent way to use medical literature is systematically to get up one subject well; to investigate it thoroughly; to trace its history and follow its development; to scrutinize diverse observations, review conflicting opinions and weigh different conclusions. Having once mastered the thing, it will be surprisingly easy to follow it through the succeeding years, for the annual increment to any given subject in medicine is astonishingly small. After one topic is exhausted another may be attacked, and so onward.

This method easily falls in with a second good one, namely, to read up fully on cases in hand. Note that I say "fully." Hastily to look up an ointment for eczema or to consult a text-book or two on the diagnosis of iritis may be one of the exigencies of practice; it is not reading. Likewise hunting up a remedy for an obscure case may possibly be a necessary makeshift, but generally it does the patient little good and the physician less. We should read for a perfect understanding of the case, which means a complete comprehension of the subject. No case can properly be considered alone. It is always in relation to variant cases under diverse circumstances. For no patient can there be a paragraph explanation and no recipe treatment.

Now, these two plans of reading naturally lead up to a third—reading to write. Of course, this plan is good only when the writer compels himself to produce really good stuff. This proviso fulfilled, I know of nothing more wholesome than the writing of papers and the reading of them where others may pass judgment. If the first two plans have been well carried out, it is reasonably certain that this one will not miscarry. The man who has excellently well worked up any subject or any case of any abnormal condition, is not only well prepared for the next one of the kind, but he is in a position to tell others something they do not know.

Pursuance of these three plans of reading will almost inevitably produce a most desirable habit,—viz., the keeping of case records. The virtues of this practice are many. Two of them are that it stimulates reading and enhances its value. Accurate comparison of our experience with that of others not only serves to impress the facts, but ripens knowledge into wisdom.

The fourth and last suggestion for medical reading is really but a summary of the other three. It is that we acquire the mental attitude, or aptitude, or habit of reading for reproduction. To be a student is not enough. We must be effective students; student soldiers, if you please, preparing for action. There is a vast difference between the acquisition of knowledge as a mere accomplishment and as a means of accomplishment. It is well for us to regard our store of knowledge not as simply an interesting museum of Nature's wonders marvellous to contemplate, but rather as an armamentarium; an orderly array of goodly weapons ready for instant use.—*The Jour. Amer. Med. Assoc.*

Pathogeny of Osteomalacia or Senile Atrophy.[1] By Eugene S. Talbot, M.S., D.D.S., M.D., LL.D., Chicago.

The fact that pathology at most implies a disturbance of balance which causes a conflict of physiologic processes is nowhere more evident than in osteomalacia. The current error which assumes that any process aiding disease must be innately nosologic interferes with diagnosis and treatment alike. The clinical and pathologic work done on osteomalacia has been vitiated by the view-point just mentioned. Osteomalacia occurs from so many nosologic states as to indicate that it arises from a process physiologic in character, but perverted to nosologic ends by anything which disturbs the balance struggle for existence between the structures.

Bones do not grow in the ordinary sense, since the bone-cells can not multiply. Apparent growth of bone is caused by destruction of bone already formed and by production of new bone. The production of new bone is one, as Minot (Embryology) points out, first to degeneration of the ossifying cartilage. Cartilage begins to be differentiated earlier than any of the mesenchymal tissues except the blood-vessels, and perhaps the smooth muscle-cells. Cartilage undergoes a degenerative change preparatory to ossifying. This is one of the many instances in the embryo where degeneration of a particular structure is necessary for advance of the body as a whole. There are, as Minot points out, two stages in the life-history of cartilage. The first (in which the cells are large) is the earlier stage, and represents the maximum of development, while the second (in which the cells are shrunken and fatty) represents a

---

[1] In "Interstitial Gingivitis, or So-called Pyorrhœa Alveolaris," I called attention to a form of bone absorption,—osteomalacia or senile absorption. Very little was said at the time, for the reason I wished to do more research work before bringing the subject before the profession. That paper was read before the Chicago Academy of Medicine, December 12, 1899.

later stage with more or less degeneration. In what is called ossification of cartilage (an erroneous term) the cartilage undergoes complete degeneration and disappears. Bone is derived always by direct metamorphosis of embryonic connective tissue or of embryonic cartilage and of periosteum. Bony tissue, as already remarked, does not grow except by additions to its surface. To a certain extent it depends on a balance between the metamorphosis of embryonic connective tissue, the formation of cartilage, and the function of the osteoblasts, which build up, and the osteoclasts, which break down.

These four conditions occur in fracture. The tissue around and between the bone ends is provisional callus. The periosteum forms the external callus and medullary tissue the internal callus. Ossification of internal callus is performed by the osteoblasts, which develop, and osteoid tissue, that later by calcic deposits undergoes a change into true bone. This bone formation is often preceded by tissue of the embryonic connective type. The osteoclasts absorb bony substances in excess. Imperfect work by the osteoblast or excessive formation would reproduce in a fracture the condition of tissue which occurs in osteomalacia. Osteomalacia hence depends on the removal of inhibitions on the physiologic balance between formative and destructive functions.

Inhibitions are exercised through the nervous system. It is not surprising, therefore, to find fully developed osteomalacia connected by many links with trophoneuroses, in which similar local bone changes occur. Prominent among these are paretic dementia and locomotor ataxia. Various changes of the bones and joints, as J. G. Kiernan [1] has pointed out, occur in paretic dementia, either in the direction of osteomalacia, of premature and excessive ossification, or hydroarticuli (thickening of the articular extremities of the long bones). Similar conditions were found previously by Charcot, Ball, and J. K. Mitchell in locomotor ataxia.

In other conditions, where like though lesser disturbances of the physiologic balance of the struggle for assimilable nutriment occur, osteomalacia and its converse likewise develop. In pregnancy such conditions are present. So far as the woman is concerned, pregnancy, as Harriet Alexander [2] has shown, is a patho-

---

[1] Journal of Nervous and Mental Diseases, 1878, p. 253.

[2] Pediatrics, January, 1901.

logic disturbance of balance hitherto existing in the organism. In consequence, nutrition and assimilation are increased, while elimination is decreased. In pregnancy, therefore, occurs an autointoxication which may express itself in major phenomena like eclampsia, or minor phenomena like the destruction of the teeth. From the influence of this last type exercised on bone growth occur not only trophic disturbances, like osteomalacia, but also, as Rokitansky[1] long ago demonstrated, osteophytes (of the cranial bones in particular). This condition, as Ducrest has shown, appears and disappears under pregnancy. While Hohl and Virchow have claimed that this condition bears merely a coincidental relation to pregnancy, corroboration of its frequency by French, German, and Italian pathologists demolishes this criticism.

Osteomalacia (the halisteresis ossium of Kilian) consists anatomically of an osteitis and periosteitis, in which the perfectly hard bones are decalcified and replaced at first by lamellar connective tissue; finally this passes centrally into the round granular medullary cell. The medullary spaces and Haversian canals increase in size, the bone corpuscles partly disappear, but in part become shorter and their processes smaller. The more complete the substitution of connective tissue the more flexible the bones become. In osteomalacia corea they are, as Winckel[2] remarks, almost as yielding as wax and not soft enough to be cut. In fully developed osteomalacia, therefore, the cartilage formation has been replaced by connective tissue.

Winckel has shown that the conditions under which puerperal osteomalacia develops are essentially those causing degeneration. By improvement of hygienic surroundings of Bavarian peasant women, his father was able to lessen the amount of osteomalacia. It does not, therefore, form a single nosologic species. It is clearly connected with the trophic factors regulating bone growth, bone repair, and bone existence. In its essence it is, like cancer, a reversion to embryonic conditions. While to some extent lower, the conditions found in osteomalacia are essentially those of the immature sea-squirt in its prevertebrate period. It is a general law of biology that structures in certain parts of an organism retain for the benefit of that organism lower characteristics. This being the case, there

---

[1] Thèse de Paris, No. 1, 1844.

[2] Text-Book of Obstetrics (American edition), p. 472.

should be one structure in the body which would give a clew to the etiology and early pathology of osteomalacia. Such a structure is the alveolar process. This is situated on the superior border of the inferior maxilla and on the inferior border of the superior maxilla. While usually considered a part of the maxillary bones, the alveolar process should be considered separately. Its structure, embryology and functions differ completely from the structure and functions of the maxillary bone. The alveolar process is composed of soft, spongy bone of a relatively cancellous structure. As early as the eleventh week of intrauterine life calcification of the deciduous teeth commences, and by the twelfth week calcic material is quite abundantly deposited. The alveolar process, being soft and spongy, moulds itself about the sacs containing the crowns of the teeth and along their roots after their eruption, regardless of position in the jaw. While the alveolar process has grown rapidly, it has developed up to this time just enough to cover and protect the follicles while calcification of the jaw proceeds. When the crowns have become calcified and the roots have begun to take in calcic material, absorption of the border of the process takes place in the order of eruption of the teeth. When the teeth have erupted, the alveolar process develops downward and upward with the teeth until it attains the depth of the roots which, in most instances, extend into the superior maxillary bones in the anterior part of the mouth, and the upper and lower teeth rest at a point in harmony with the rami. The depth to which they penetrate depends on the length of the roots and the alveolar process, and this in turn depends on the length of the rami. The incisive fossa, the cuspid eminence and fossa give evidence of this externally. The sockets are lined with extensions of the process, thus making its upper border irregular.

When the temporary teeth are shed, the alveolar process is absorbed to make room for the eruption of the permanent set. The crowns of these, being larger than those of the temporary teeth, require more space and the process must enlarge to accommodate them. It then is rebuilt about the roots of the teeth on a much larger scale. When the temporary teeth are lost the alveolar process is reabsorbed. It is hence developed twice and absorbed thrice, provided the second set of teeth is lost. The process is a very thin, unstable structure, naturally well nourished with blood-vessels. As the skull and brain are gaining in the struggle for existence between the face, jaws, skull, and brain, the jaws with the alveolar process must decrease in size with advance. This fact and the changes just described render the process a doubly transitory structure. For this reason it is very susceptible to metabolic changes, to mineral and vegetable drugs and poisons, as well as to changes in temperature and climate. This is, in part, due to the readiness with which checked elimination elsewhere finds exit through the mouth and nose. The great supply of blood-vessels in the alveolar process plays a part in determining elimination.

Should man live long enough, and should the physiologic process of involution set in, his second set of teeth would disappear as a consequence of osteomalacia of the senile atrophy type. The lower vertebrates are called *Polyphyodontia,* because there is a continuous succession of teeth, not a separation into two sets. In some mammals this condition persists. The pachyderms and rodents (which are connected embryologically) present phenomena analogous to that of the *Polyphyodontia.* In the rodents, especially the nut-eating rodents, continuous growth occurs in the incisors as they are worn down. Should one of the incisors disappear, the opposing one so grows as to interfere with the gnawing powers. Many a squirrel has thereby lost its life. In the elephant not more than three teeth are in use at a time. Those worn down are shed, while new teeth are added.[1] Thus the whole number of teeth are not in place at one time. In other pachyderms, like the hyrax, similar conditions are found. Among the edentates, tooth conditions form a natural transition to the *Sauropsidæ* and *Icthyopsidæ.* A curious link also occurs in the *Monotremata,* where the duck-bill has deciduous teeth during youth, which are afterwards absorbed to make way for horny plates. Judging from the conditions found in the toothed birds, the same result occurred at a phase in evolution of toothed birds from reptiles. In man, however, this degenerative process (involving absorption of the alveolar process and loss of the teeth) is continuously present in a latent way. The alveolar process is, therefore, more subject to change from altered metabolism, due to trophic disorders of nutrition than other structures. Osteomalacia or senile absorption occurs with greater rapidity, and produces more decided change in the alveolar process than in other bones. Causes which would not affect bone structure elsewhere markedly derange it.

---

[1] Tomes's Dental Anatomy, p. 405.

While osteomalacia may affect the alveolar process at any period of life after the eruption of the first set of teeth, it does not usually occur until the period between twenty-five and thirty-five. Before this the osseous system is in its constructive state and lime salts are being deposited rapidly. Later in life the constructive stage is complete, and material sufficient only to repair waste is deposited. At the periods of stress metabolic changes are most active,—during puberty and adolescence (fourteen to twenty-five), during the climacteric (forty to sixty), when uterine involution occurs in women and prostatic involution in men, and, finally, during senility (from sixty upward), when the disease is always present to a greater or less degree. While in allied conditions men are most influenced in this disorder, the sexes seem to be affected about equally. Here the influence of pregnancy comes into play. Pregnancy disturbs the physiologic balance hitherto existing, especially along the line of assimilation and elimination. The well-known dental efforts of pregnancy (whose underlying cause affects the alveolar process) are due to this factor. This is purely a constitutional affection.

Among the causes are non-elimination of toxic substances, whether due to autointoxication, to bacterial action, or to metallic and vegetable drugs. Disorder or disease of any excretory organ (kidneys, bowels, skin, or lungs) will produce the most marked effect, first, on the constitution of the blood, and secondly, on the alveolar process, with resultant osteomalacia.

The urine, as Bouchard has shown, contains each day in a normal individual sufficient toxins to cause death if not excreted. This condition is markedly increased after prolonged nervous explosions, like those of epilepsy or hysteria. This was pointed out thirty years ago by Meynert, who demonstrated that the status epilepticus (condition of rapidly recurring convulsions) was due to the accumulations of a proteid body in the system. The status epilepticus is preceded by a decrease in toxins in the urine and succeeded by an increase. This is likewise true as to the influence of non-elimination by the other excretory organs (bowels, lungs, and oral cavity), as well as to the non-exercise of its poison-destroying power by the liver. Non-elimination, moreover, interferes with ordinary digestive functions, and hence increases its own extent. Another factor in autointoxication is production of toxic products in such quantity as to prevent destruction by organs like the liver and consequent elimination, since a product to be properly eliminated must be changed to a particular chemical type. Among the factors which affect both these elements of elimination is the power over growth and repair exercised by the nervous system. In part, this influence is exerted through control of blood-supply by the vasomotor nervous system and in part by that direct control of the nervous system over tissue change, which is known as its trophic function.

Both influences are affected by nervous strain. Sudden emotion may, as Bichat demonstrated decades ago, produce marked defects on bile secretion and may occasion jaundice. Cases are far from infrequent in which emotions like jealousy produce a mimicry of gall-stone colic in neuropaths. Murchison, Christison, and Thompson have traced attacks of biliary colic to jealousy. Other liver changes from sudden nervous disturbance, whether of mental type or not, are not rare. As mental impressions are communicated to the central nervous system purely through mechanical changes in the nerves, such influence must be purely material in operation. As the brain exercises a checking influence on the operations of the liver, these mental influences produce two effects. The mental shock increases the checking action of the central nervous system on the local ganglia of the liver and destroys the checking action of the liver ganglia, and in consequence these go too fast, resulting in their exhaustion. Either of these conditions interferes with the poison-destroying action of the liver, and accumulation of waste products is the result.

What is true of the liver is true of the other organs. This is especially noticeable, as Tuke points out, in regard to the kidneys. The action of mental anxiety or suspense in causing a copious discharge of the pale fluid is familiar enough to all, especially to the medical student about to present himself for examination, the amount being in a pretty direct ratio to his fear of being plucked. The frequency of micturition may, however, arise from nervous irritability of the bladder without increase or even with diminished secretion. Still, the action of the skin is usually checked, the extremities are cold, and the kidneys have to pump off the extra amount of fluid retained in the circulation. Elimination of the substance usually separated from the blood is diminished, as compared with the aqueous character of the whole secretion. The odor may be affected by the emotions in man as in animals. Prout is of the opinion that mental anxiety will produce not only non-elimination, but also change in the chemical character, as indicated by odor and otherwise. Disturbances in the medulla produce, as Claude Bernard long ago showed, a markedly pale, excessive urine. These disturbances often arise from intellectual strain or emotional shock. The influence of emotional states on secreting processes and thereby indirectly on autointoxication states, is illustrated in the fact long ago pointed out by Tuke that pleasurable emotions increase the amount of gastric juices secreted, the opposite effect being produced by depressing passions. Beaumont found in a case of gastric fistula that anger or other severe emotions caused the gastric inner or mucous coat to become morbidly red, dry and irritable, occasioning at the same time a temporary fit of indigestion.

The influence of fear and anxiety on the bowels is as well marked as that on the bladder and kidneys. Apart from muscular action, defecation may become urgent or occur involuntarily from various causes. The increased secretion from the intestinal canal may occur from fear, and in some cases from the altered character of the secretion itself. While in this respect the influence of fear may be inconvenient in man, it naturally assists escape in some animals, as the skunk.

Emotions powerfully excite, modify, or altogether suspend, as Tuke has shown, the organic functions. This influence is transmitted not only through the vasomotor nerves, but through nerves in close relation to nutrition and secretion. When the excitement is of peripheral origin in sensory or afferent nerves, it excites their function by reflex action, so that as emotion arises it may excite the central nuclei of such afferent nerves, and this stimulus be reflected on the efferent nerves, or it may act directly through the latter. Pleasurable emotions tend to excite the processes of nutrition, hence the excitement of certain feelings may, if definitely directed, restore healthy action to an affected part. Violent emotions modify nutrition. Various forms of disease originating in perverted or defective nutrition may be caused primarily by emotional disturbance. Emotions by causing a larger amount of blood to be transmitted to a gland increase sensibility and warmth, and stimulate its function or directly excite the process by their influence on nerves supplying the glands. Painful emotions may modify the quality (*i.e.*, the relative proportion of the constituents) of the secretions.

Imperfect elimination of effete matter from the lungs is a fruitful source of autointoxication. The more marked forms are those of tuberculosis, in which there is great debility and in which there is greater waste than repair. Self-poisoning is continually going on, and will continue until death. The chest capacity for the inhalation of pure air is almost *nil*, hence the blood is improperly oxygenated and soon ceases to convey nutriment to the tissues. Eighty per cent. of criminals who die of tuberculosis in prisons have undeveloped chest walls. Degeneracy, therefore, cuts quite a figure in the rôle of autointoxication. Degenerates with contracted chest walls are, however, more frequently found. Many undeveloped individuals in every walk of life for this reason have tuberculosis. People with undeveloped chest walls and chest capacity may not have tuberculosis and yet may suffer from autointoxication. Those who have had pneumonia with adhesion, and who are thus unable to oxygenate the blood, are subject to this disease. Asthmatics and hay-fever patients suffer from autointoxication and alveolar absorption. When the skin is overstrained as to excretion through the kidney and bowel overstrain, the lungs are forced to take on increased work with imperfect oxygenation as a result. This is noticed in the odor of the breath in Bright's disease and in the air-hunger of diabetes, etc. In nerve-strain states and in the condition described by Albu, not only do excretory organs suffer, but the secretions of those glands, like salivary and buccal glands, are so altered as to become irritants. These excretory conditions not only result on autointoxication states, but are modified by trophic nerve function alterations. By trophic changes are meant such tissue alterations as occur in morbid conditions from disordered function of the centres of nutrition. Peripheral as well as central may be involved. The well-known law of Wallerian degeneration of nerve-fibres is an illustration, the posterior spinal ganglion acting as a trophic centre for the fibres of the posterior root in the cord itself. Trophic action may, therefore, be peripheral, though in extensive changes, as a rule, central (cerebral or spinal) origin should be looked for.

The constitutional result of acute and chronic infections and contagions is apt to be an autointoxication plus the action of the germ toxin. All the exanthemata have at times been followed by wasting or necrosis of the alveolus. Here the condition is notably symmetric, and accompanied by disorders of the osseous system elsewhere. The same is true of la grippe and tuberculosis. The well-marked disorder known as Riggs's disease has been charged by Peirce, Kirk, Rhein, Robin, and Magitot to the direct influence of an arthritic state (gouty and rheumatic), and regarded as a special type of arthritic manifestation. The alveolus is clearly vulnerable to the toxins of many infections. It is likewise quickly affected by some autotoxic influences from disordered metabolism. Its vital resistance to these agencies is less than of other tissues. It is the earliest sacrifice when these or any toxins disturb the harmony of the organism.

A cause other than the action of toxins exists for implication of these parts. Whenever tissue waste, whether local or general, exceeds repair there is trophic change. This latter depends directly on disordered local or general nervous functions. Trophic alterations from the first cause appear in growth disorders of the nails and loss of hair (alopecia) after fevers, the most familiarly obvious examples of this pathologic process. Of the other type are localized atrophies, where the direct intervention of toxins can be excluded. The alveolus is liable to the first form of trophic deterioration. The influence of acute diseases on the alveolus is probably thus exerted in many cases rather than by direct infection. Where no cause has been ascertained, examination directed to this factor would probably reveal it. The general failure of the trophic centres after the prime of life (in senile states), which is attended with loss of teeth and wasting of the alveoli, is the most obvious instance of trophic failure affecting the part. Even simple anæmia may thus give rise to alveolar wasting.

The more marked forms of constitutional disorders (typhoid fever, pneumonia, tuberculosis, syphilis, indigestion, pregnancy, etc.) produce intense results.

The second form of trophic failure in the alveolus is less prominent, since it generally coexists with overshadowing disturbance elsewhere, which it creates to a certain extent. Cruveiller noticed its occurrence associated with simple paraplegia, regarding it as of nervous causation. In facial hemiatrophy, local wasting of the alveolus has appeared before the disorder has involved the jaws generally. This is sometimes due to a local cause, but its occurrence and association with other neurotrophic symptoms are suggestive.

The causes which act locally to produce direct autointoxication are the toxic effects of mercury, lead, brass, uric and other acids, potassium iodide, and allied agencies, acting in a similar manner to scurvy. While it is not the intention to discuss at length the toxic action of these substances, a case may be cited in illustration of their similarity of action and results on the tissue. Garnier and

Simon have observed the case of a boy suffering from an obstinate enteritis. Milk was found to disagree, so a purée of vegetables and chopped meat was given. The boy improved for a while, but hæmatogenous jaundice occurred. On investigation the jaundice was found to be due to the action of lead on the liver, the lead having been introduced into the food through a meat-chopper. In this case, the usual symptoms of lead poisoning were absent, but through its action on the liver jaundice had appeared. Scurvy produces the same train of symptoms as the metals, through its disturbance of the metabolism.

The jaws of the hereditarily defective, whether defect be in the direction of advance or degeneracy, are fruitful soil for the development of osteomalacia. In the mouths of the congenital, deaf, dumb, blind, feeble-minded, and delinquent children, osteomalacia attacks the alveolar process before the osseous system has reached its growth. Here, as a consequence of trophic change, metabolic action and premature senility, osteomalacia may occur with the first set of teeth at two years, or at any period thereafter. This may be called juvenile osteomalacia. Regulating teeth and senile absorption are predisposing causes to osteomalacia.

Osteomalacia of the alveolar process is almost as common among domestic and wild animals in captivity as it is in man. Wild animals in zoologic gardens without proper exercise, in close confinement, with impure air, and fed on too easily digested food, naturally acquire autointoxication resulting in osteomalacia. This is particularly noticeable in monkeys, whose changes of environment render them very susceptible to disease, especially tuberculosis. Trophic changes and impaired metabolism are thereby so impressed on monkeys that not infrequently the first teeth become prematurely loose and drop out. The horse and cow are prone to this disease. Cattle returned to the stable after a summer's sojourn in the field, and then being fed on a changed diet without the usual exercise of cutting grass with their teeth, undergo a reaction in their jaws, and osteomalacia results. "Cribbing" of the horse is a marked illustration of the uneasy feeling resultant on this reaction. Cattle fed on brewers' grain and slop suffer most. Dogs afford the best opportunity, however, for studying inflammation and osteomalacia among animals. Nearly every dog in the dog hospital suffers with this disease; twenty-five per cent. of roving curs at four years of age have the disease; eighty per cent. of eight-year-old, at least ninetyfive per cent. of twelve-year-old, and all fourteen-year-old dogs have the disease. House dogs suffer to a marked extent with osteoma-

FIG. 1.

lacia of the alveolar process, no doubt from being trained to house cleanliness, which interferes with natural excretion, causing auto-intoxication and odor.

FIG. 2.

The following models show the action of osteomalacia on the jaws and teeth: Fig. 1 is that of a physician thirty-six years of age. Fig. 2 is that of a physician thirty-eight years of age. Both of these gentlemen are apparently in the best of health. One has slight indigestion, which is the cause of absorption; the other took calomel for malaria fifteen years previous, this being a predisposing cause. In each case all the teeth are involved both inside and out. Some of the teeth are becoming loose. There is no pus in either case. The gums are apparently healthy.

FIG. 3.

In consulting the literature on the subject, I find that absorption of the alveolar process and recession of the gums have always been attributed to the severe use of the tooth-brush. There are certain conditions in which the tooth-brush will assist absorption of the alveolar process. These are easily observed. I refer to the position of the cuspid teeth, where they stand prominently and are the most conspicuous part of the alveolar process. The bone over the roots is as thin as tissue-paper, and the slightest friction causes a low form of inflammation, which in turn produces absorption of the bone, exposing the root. The brush never, however, produces senile atrophy in other parts of the mouth.

The absorption of the alveolar process in osteomalacia is not always uniform, as sometimes only one or two teeth are involved,

Local conditions modify the extent of the disease. In most cases, however, there is a gradual absorption of bone about all the teeth.

The pathology of this disease about the teeth is not unlike that of osteomalacia of the pelvis, spine, and other bones of the body, as demonstrated by Hektoen, halisteresis being the principal form of absorption. Perforating canal absorption, as described by Volkmann, is very common, passing through fragments of bone. Lacunar absorption is also present, and osteoclasts are frequently found. Howship's lacunæ, containing osteoclasts, are found in the margin of irregular islands of bone. This form of absorption,

Fig. 4.

while not always present, does not cut so much figure as halisteresis, it being much slower in its action. New osteoid tissue is rarely ever seen, since this absorption is a natural destruction of bone and is never reproduced.

Here, then, is the basic explanation of interstitial gingivitis or so-called pyorrhœa alveolaris—osteomalacia or senile absorption is the underlying basis of this disease.

The preceding illustrations represent the alveolar process of a man forty-eight years of age, killed in an accident. The teeth and bone decalcified in the usual way were prepared for the microscope. Fig. 3 shows four areas of bone absorption called halisteresis (melting away of bone substance). The waste products become irritants in the blood-stream, and set up a low form of inflammation in the Haversian canals. The inflammation thus set up produces rapid absorption. Each of these local areas enlarges until they join. In this way large areas are produced. In the centre of this illustration is seen an Haversian canal with active inflammation around it. The bone is absorbed. The inflammatory process is in the trabeculæ or fibrous part of the bone. Adjoining is a large area with bone absorption, but the fibrous part of bone remains unbroken. The inflammatory process is seen throughout. At the lower border of the picture are two large areas of bone absorption. The trabeculæ

FIG. 5.

are seen, with round-celled infiltration, while the centre is destroyed. At the right absorption and destruction of the trabeculæ are seen to the margin of the bone.

Fig. 4 shows halisteresis at two Haversian canals. One area is much larger than the other. Both have met, and the area of inflammation will be much enlarged. The trabeculæ are present and filled with round-celled infiltration.

Fig. 5 illustrates a large area of absorption with destruction of the fibrous tissue to a larger extent. Around the border is seen a small amount of inflamed fibrous tissue. An artery, once an

FIG. 6.

FIG. 7.

Haversian canal, is also seen. About the large area are also seen three Haversian canals with the inflammatory process just beginning.

Fig. 6 shows four centres of absorption at Haversian canals. Through the picture may be seen dark lines running in all directions. These are vessels of Von Ebner, through which Volkmann's canal absorption takes place. A beautiful illustration of this is the canal running from one large area of absorption to the other.

Fig. 7 shows the third form of bone absorption,—lacunæ or osteoclast absorption. Here a large area of bone is destroyed by these large cells.

Fig. 8 is a low power, showing the distribution of the alveolar

Fig. 8.

process between the roots of two teeth. Very little of the bone remains. When the trabeculæ or fibrous tissue is destroyed in large areas, and especially in transitory structures, it is rarely restored.

Does it not seem reasonable, therefore, that the etiology of osteomalacia of the pelvis and other bones of the body is the same as that of the alveolar process, since the pathology is the same, namely, faulty metabolism and elimination, autointoxication, and drug poisoning?

A relationship exists between Dercum's disease (adiposis dolorosa) and osteomalacia, according to Pennato,[1] who finds several cases on record in which changes in joints occurred. Bone nutrition is regulated by the trophic centres markedly affected in Dercum's disease. A case of a thirty-five-year-old woman, observed by Pennato, was that whose first adiposis appeared at twenty-three. She slowly developed adiposis dolorosa and lost her teeth, except the inferior cuspids and one molar, although caries did not occur. Symptoms referable to the bones appeared almost at the outset, consisting of distortion of the right knee, curvature of the leg, some years later fracture of a clavicle, and still later of the left humerus. When Pennato saw the case the legs and thighs were semiflexed. Complete extension was impossible on the right side, on account of rigidity in the knee, with partial dislocation of the tibia inward. Such cases bear out the position that autointoxication is the initial cause of osteomalacia, since in obesity or lipomatosis the products of autointoxication are always present in the alveolar process, and since Dercum's disease is an exaggeration of the nutritive degeneracy which tends to appear at the second dentition.[2]

RÉSUMÉ.

1. Osteomalacia may and does exist for years in pelvic and other bones before the symptoms can possibly be recognized by the physician or surgeon.

2. The object of this paper is to show that osteomalacia can be studied earliest in the alveolar process.

3. The alveolar process is the most transitory structure in the body. It develops twice, and is absorbed thrice if the second set of teeth are shed. The evolution of the face, whereby the jaws are decreasing in size, with the many complications thereon resultant, renders the jaws and alveolar process increasingly transitory.

4. In the evolution from the lowest vertebrates up there has been a continuous succession of teeth (polyphyodont), as found in some selachians; a partial continuous succession, as in some mammals; and a comparatively permanent set of teeth, as in man. This shedding of teeth, due to a process called senile absorption, atavistic in type, takes place in every one to a greater or less extent

---

[1] Medical Bulletin, April, 1904.

[2] Kiernan, Alienist and Neurologist, 1900.

after forty-five years of age. Should man live in a comparatively healthy state long enough he would lose all teeth from this process.

5. Degenerate children from precocity, due to arrested development at the senile or simian period of intrauterine life, may show symptoms of this disease in connection with the first set of teeth at from six to ten years of age. A monkey which died of tuberculosis at one year had osteomalacia, which exposed the roots of all the temporary teeth, while three had dropped out.

6. Constitutional causes like autointoxication and drug poisoning are the etiologic factors. Even the mildest types of autointoxication, due to indigestion, change in climate from hot to cold, and *vice versa,* with corresponding change in food, giving more work to some eliminating organs and less to others, as well as to mild forms of drug poisoning, may be potent in this particular.

7. The effect of autointoxication and drug poisoning is first irritation through blood-streams, often causing endarteritis obliterans. Since the arteries are terminal, irritation readily causes inflammation and halisteresis.

8. Osteomalacia is as common among wild animals in captivity as in domestic animals.

9. The influence of bacteria as a cause has not been demonstrated by Koch's law.

10. If due to autointoxication, the effete matter should be removed from the system.

11. Osteomalacia or senile atrophy is the basic explanation of interstitial gingivitis, or so-called pyorrhœa alveolaris. Will not the same line of reasoning hold true of osteomalacia of the bones of other parts of the body?

## Figures and Tables

**Fig. 1. f1:**
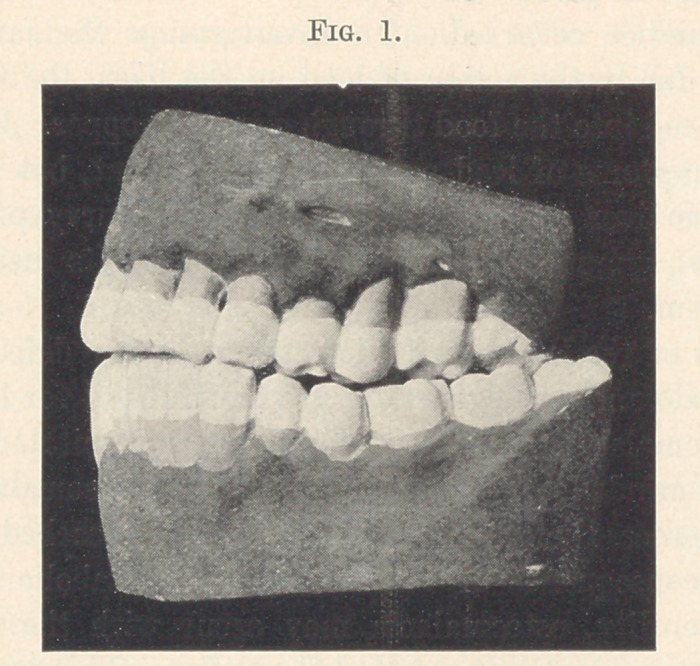


**Fig. 2. f2:**
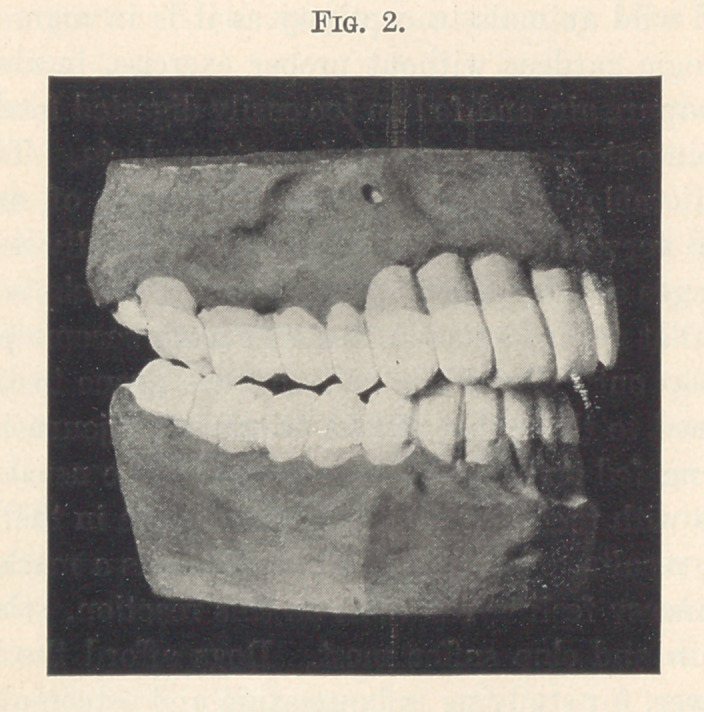


**Fig. 3. f3:**
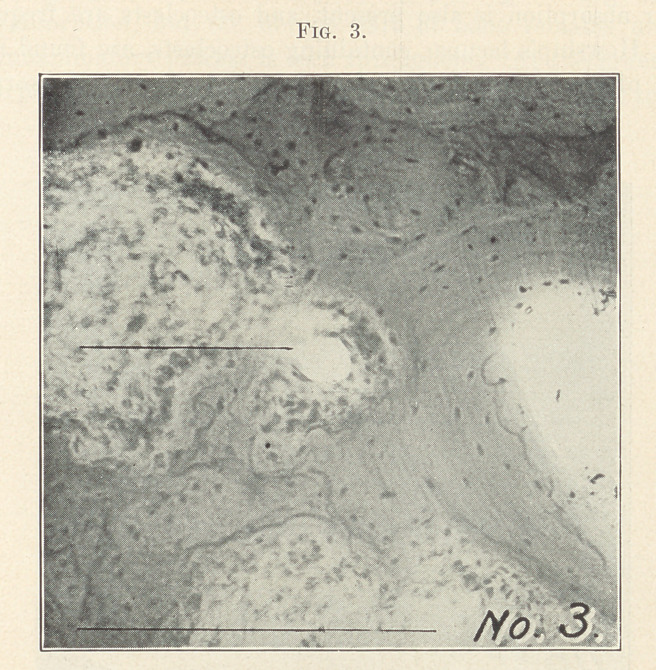


**Fig. 4. f4:**
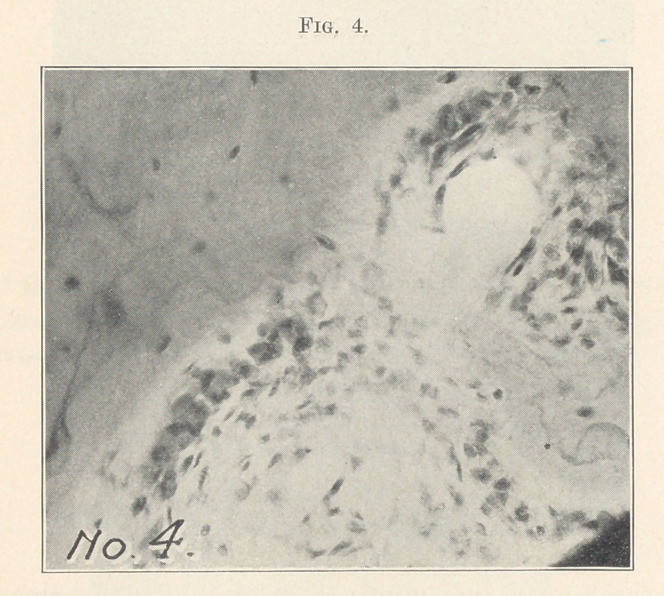


**Fig. 5. f5:**
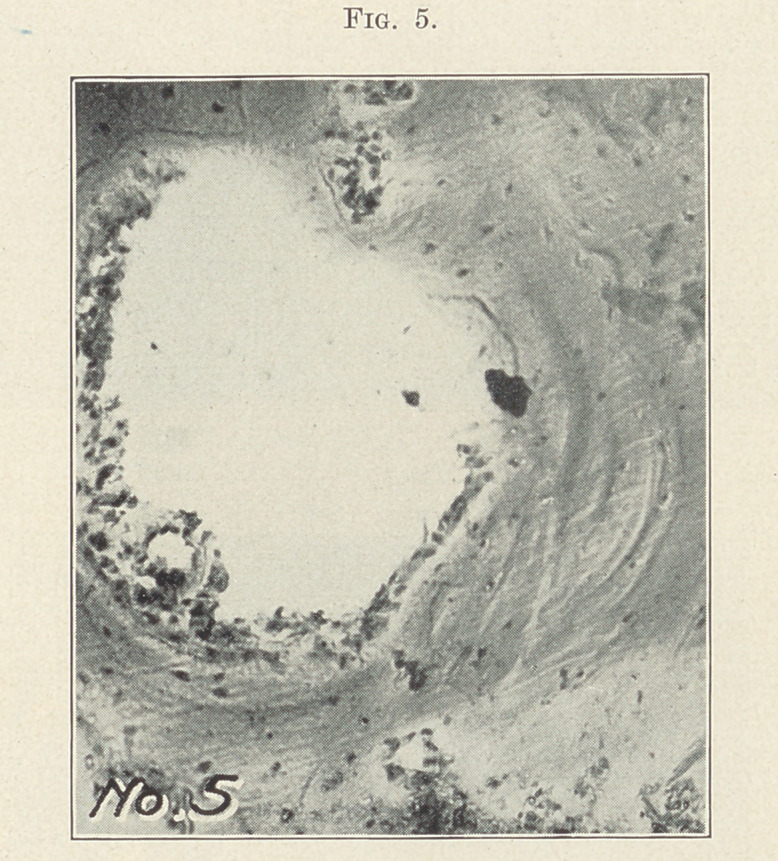


**Fig. 6. f6:**
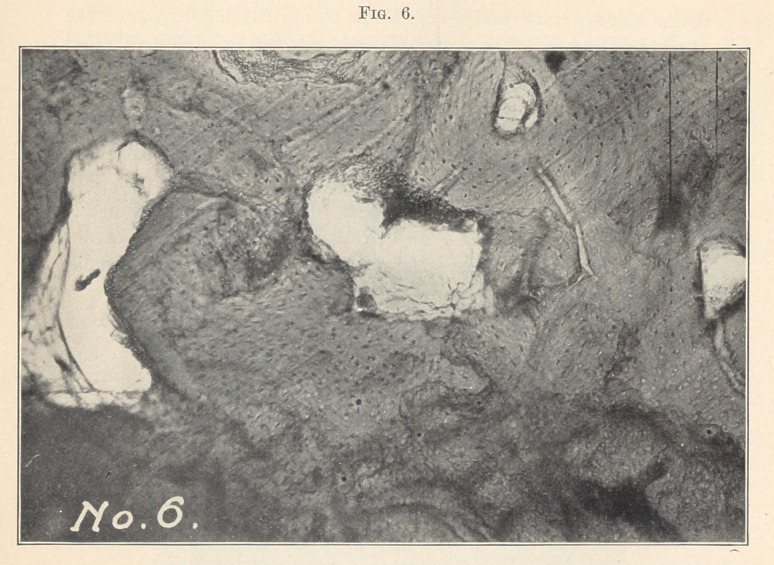


**Fig. 7. f7:**
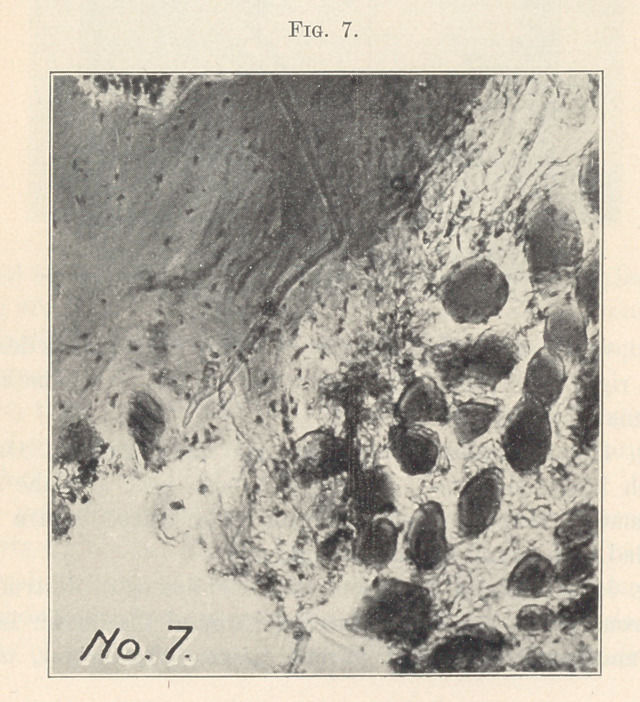


**Fig. 8. f8:**